# Discovery of SIRT7 Inhibitor as New Therapeutic Options Against Liver Cancer

**DOI:** 10.3389/fcell.2021.813233

**Published:** 2022-01-31

**Authors:** Chen Zhang, Yaqi Li, Bohao Liu, Chao Ning, Yimin Li, Ying Wang, Zhuan Li

**Affiliations:** ^1^ The Key Laboratory of Model Animals and Stem Cell Biology in Hunan Province, The Key Laboratory of Study and Discovery of Small Targeted Molecules of Hunan Province, and Department of Pharmacy, School of Medicine, Hunan Normal University, Changsha, China; ^2^ The National and Local Joint Engineering Laboratory of Animal Peptide Drug Development, College of Life Sciences, Hunan Normal University, Changsha, China

**Keywords:** virtual screening, SIRT7, histone deacetylase, HCC, therapy

## Abstract

Optimal therapeutic strategies for liver cancer patients remain challenging due to the high recurrence rate after surgical resection and chemotherapy resistance. Emerging evidence has shown that epigenetic factor SIRT7 is involved in various aspects of cancer biology, while inactive SIRT7 reverses human cancer phenotype and suppresses tumor growth. In the present study, we predicted the SIRT7 structure by using the fold recognition (or threading) method and performed structure-based virtual screening to develop specific SIRT7 inhibitor by docking 939319 structurally diverse compounds with SIRT proteins. Compounds with high affinities to SIRT7 but low affinities to other SIRT proteins were chosen as candidates of specific SIRT7 inhibitor. Our leading compounds 2800Z and 40569Z showed strong interaction with SIRT7 protein, and specifically inhibited SIRT7 deacetylation activity *in vitro*. Our docking results also revealed that ARG-120, TRP-126, and HIS-187 were critical sites responsible for interaction of SIRT7 with small molecules. Mutations in the aforementioned sites significantly abolished interaction and inhibitory effects of compounds to SIRT7. In addition, *in vivo* data indicated that compounds 2800Z and 40569Z were able to induce apoptosis and increase chemosensitivity to sorafenib in human liver cancer. Our findings demonstrated targeting SIRT7 may offer novel therapeutic options for cancer management, and the value of compounds 2800Z and 40569Z as chemical probes for the study of SIRT7 biological functions as well as starting leads for the development of new therapeutic options against liver cancer.

## Introduction

Liver cancer ranks sixth in all cancer incidence and third in cancer motility worldwide, with more than 900,000 new cases reported in 2020 (Siegel et al., 2020; Sung et al., 2021). Age-specified studies have also revealed that liver cancer incidence among young persons is significantly increased in recent years (Miller et al., 2020). Despite great efforts having been made to improve diagnosis of liver cancer, a large portion of patients are still diagnosed at an advanced stage due to lack of signs and symptoms of this disease at an early stage (Heimbach et al., 2018). Advanced liver cancer is possibly the most aggressive cancer type which remained clinically challenging to manage due to the low responsiveness to therapy and the high recurrence rate (Bruix et al., 2021). While systemic radiotherapy and chemotherapy are the options for advanced liver cancer, they often show very poor responses (European Association for the Study of the Liver, 2018; Heimbach et al., 2018). In more frequent instances, liver cancer is refractory to chemotherapy as it acquires drug resistance and rapidly develops intrahepatic recurrence and distant metastasis (Llovet et al., 2015; Mlynarsky et al., 2015). Therefore, there are still urgent needs to improve our understanding of the molecular mechanisms that underlie liver cancer malignancy, which will potentiate the mechanism-based translational strategy for future therapeutic development against liver cancer.

Tyrosine kinase inhibitor (TKI) including sorafenib and lenvatinib are currently available options for the treatment of advanced liver cancer that can suppress VEGFR, PDGFR, RET, and c-Kit activation, and inhibit tumor cell proliferation (Perera et al., 2020). TKIs remain the most effective single-drug therapy for liver cancer so far and improve patients’ median overall survival (OS) in clinical trials, but they only achieve modest treatment responses (Sangro et al., 2012; Bruix et al., 2021). In this regard, a combination therapy with sorafenib and immune checkpoint inhibitor has been approved as first-line therapy for advanced liver cancer, and the results indicated the progression free survival (PFS) is prolonged compared to sorafenib monotherapy (Dyhl-Polk et al., 2021). More recently, a combination of lenvatinib with immune checkpoint inhibitor is currently evaluated in a clinical trial (NCT03841201) as first-line therapy in liver cancer. These data clearly suggested the importance of combining antitumor drugs that target different signaling pathways to improve treatment efficacy and clinical outcomes.

SIRT7 is a family member of the silent information regulator 2 (Sir2) proteins that are described as NAD^+^-dependent class III histone deacetylases (HDAC III) (Barber et al., 2012). Unlike other SIRT proteins, SIRT7 is predominantly localized in the nucleus where it regulates RNA polymerase I transcription by targeting H3K18 for deacetylation (Chen et al., 2013; Song et al., 2017). Besides H3K18, SIRT7 has also been reported to target several non-histone proteins, including p53 (Vakhrusheva et al., 2008), GABP-β (Ryu et al., 2014), FOXO3 (Li et al., 2017), and U3-55k (Chen et al., 2016) for deacetylation, and has been implicated in multiple cellular functions including hepatic lipid metabolism, mitochondrial homeostasis, and adipogenesis. Emerging evidence has also implicated SIRT7 in cancer biology (Barber et al., 2012; Kim et al., 2013; Malik et al., 2015). H3K18 deacetylation by SIRT7 is important for maintaining the fundamental properties of the cancer cell phenotype and knockdown of SIRT7 influences cell cycle control, and impairs cancer cell transformation (Barber et al., 2012). Elevated expression of SIRT7 is frequently observed in many cancer types including epithelial prostate carcinomas (Malik et al., 2015), hepatocellular carcinoma (Zhao et al., 2019), colorectal cancer (Yu et al., 2014), and lung cancer (Cheng et al., 2019), and high SIRT7 levels are associated with poor prognosis. Inactive SIRT7 reverses aggressive cancer phenotypes (Barber et al., 2012; Li et al., 2019), inhibits metastasis (Malik et al., 2015), and sensitizes cancer cells to therapy (Zhang et al., 2016; Zhao et al., 2019). In prostate cancer cells, SIRT7 cooperates with SIRT1 to suppress E-cadherin regulatory genes to promote EMT, and high SIRT7 levels are associated with metastatic disease and poor prognosis (Malik et al., 2015). In HCC, SIRT7 expression is also upregulated in a large cohort of HCC patients (Kim et al., 2013), and we have identified that elevated SIRT7 expression is associated with chemosensitivity by regulating TP53 activity in human HCC (Zhao et al., 2019).

We have previously identified that elevated SIRT7 expression is associated with chemoresistance in human liver cancer, and pan-SIRT inhibitor enhances chemosensitivity to doxorubicin, which suggested SIRT7 may serve as a therapeutic target of liver cancer (Zhao et al., 2019). Here, we performed structure-based virtual screening to develop small-molecule inhibitors that specifically target the catalytic domain of SIRT7 and evaluated their anticancer effects. Our lead compounds, 2800Z and 40569Z, specifically inhibited the deacetylation activity of SIRT7, blocked proliferation, enhanced chemosensitivity of sorafenib, and reduced tumor burden both *in vitro* and *in vivo*. Our data thus strongly suggest SIRT7 represents a druggable target in human cancer and provides valuable preclinical evidences supporting compounds 2800Z and 40569Z as starting leads for the development of new therapeutic options against liver cancer.

## Materials and Methods

### Protein Structure Prediction and Analysis

Structural model of the human SIRT7 (Accession: NP_057622.1) was predicted by *I-tasser* (https://zhanggroup.org/I-TASSER/) as previously reported (Roy et al., 2010; Yang et al., 2015; Yang and Zhang, 2015). The main threading templates were human SIRT6 (PDBID:3K35) and SIR2 (PDBID:2H59). The Enzyme Commission (EC) numbers and active sites were analyzed by *COFACTOR* (https://zhanggroup.org/COFACTOR/) (Roy et al., 2012; Zhang et al., 2017) and *COACH* (https://zhanggroup.org/COACH) (Yang et al., 2013a; Yang et al., 2013b) based on the predicted structure.

### Structure-Based Virtual Screening

Structure-based virtual screening was carried out by *Autodock Vina* (Trott and Olson, 2010). The receptor grid was built up by *Autodocktools* based on the predicted active site. Chemdiv database (https://www.chemdiv.com) containing 939319 structurally diverse compounds was docked to SIRT7, and the top 100 compounds that showed high affinity to SIRT7 were chosen to further dock with SIRT1 (PDB ID:4ZZI), SIRT2 (PDB ID:5Y0Z), SIRT3 (PDB ID:4JT9), SIRT5 (PDB ID:3RIY), and SIRT6 (PDB ID:5Y2F) for evaluating candidate compounds of specific SIRT7 inhibitor. Grid box was obtained from PyMOL Plugin GetBox, and all the active site residues were selected. The centers of the grids were 44.5, 44.9, and 48.9, and the size of x was 26.9, size of y was 26.7, and size of z was 22.3.

### Molecular Dynamics Simulation

Molecular dynamics (MD) simulations were performed by *AMBER20* (D.A. Case et al., 2021). Hydrogen atoms of proteins were added by the *tleap* module based on the ff14SB (Ponder and Case, 2003) force field. The force field parameters of all the candidates were generated by the *Antechamber* module using the AM1-BCC (Jakalian et al., 2000) charge model. The systems were soaked in TIP3P (Jorgensen et al., 1983) water, and chloride ions were added to neutral. The structures were energy-minimized by 4000 steps of steepest descent followed by 1,000 steps of conjugate-gradient. All the systems were heated in the NPT ensemble from 0 to 300 K with weak restraints (force constant *k* = 10) on the proteins in 30 ps. Finally, 100 ns MD simulations with a time step of 2 fs were performed for all the systems in the NVT ensemble on 300 K. The temperature was controlled using the Langevin thermostat, while the pressure was controlled by the anisotropic Berendsen barostat.

### Binding Free Energy Analysis

The root-mean-square deviations (RMSD) of all the heavy atoms were calculated with the reference to the initial conformation. 100 snapshots were extracted every 100 ps from the last stable 10 ns MD trajectory and used to calculate the binding free energies using MM-PBSA.py (Miller et al., 2012).

### Antibodies and Chemicals

Sorafenib (BAY 43–9,006) was purchased from Selleck Chemicals (Shanghai, China), and 2800Z and 40569Z were purchased from Chemdiv (CA, United States). Anti-GAPDH (ab125247), anti-p53 (acetyl K373) (ab62376), and NOXA (ab13654)were purchased from Abcam (Cambridge, United Kingdom). PARP (9532) and cleaved Caspase-3 (9662) were purchased from Cell Signaling Technology (MA, United States). Anti-Flag (M2) was purchased from Sigma-Aldrich.

### Cell Culture, Plasmids, and Transfection

HepG2 and L02 cells were provided by Dr. Xiaoping Yang (Medical College of Hunan Normal University, Changsha, Hunan, China), and 293T cells were provided by Dr. Xiyun Deng (Medical College of Hunan Normal University, Changsha, Hunan, China). HepG2 cells and 293T cells were grown in Dulbecco’s modified Eagle’s medium (Gibco, United States) containing 10% fetal bovine serum (FBS, United States) and 1% penicillin/streptomycin (both from Gibco) at 37°C in a humidified 5% CO_2_ incubator. L02 cells were grown in RPMI 1640 (Gibco, United States) containing 10% fetal bovine serum (FBS, United States) and 1% penicillin/streptomycin at 37°C in a humidified 5% CO_2_ incubator. Flag-SIRT7 R120G, Flag-SIRT7 W126G, and Flag-SIRT7 H187 G mutants were generated by using the Q5 Site-Directed Mutagenesis Kit from New England BioLabs (Ipswich, MA). Cells were transfected in the serum-free medium (Opti-MEM, Invitrogen) by using X-tremeGENE (Roche, United States) according to the manufacturer’s instructions.

### Western Blotting

Whole-cell lysates were prepared from cells that had been washed and harvested by centrifugation in PBS. Cell pellets were resuspended in RIPA buffer that contained 50 mM Tris, pH 7.5, 150 mM sodium chloride, 1% NP-40, 0.2% SDS, 0.5% sodium deoxycholate, 0.1 mM EDTA, and 1% protease and phosphatase inhibitors (Sigma-Aldrich). Lysates were centrifuged, and supernatants were collected. Centrifugation of cell lysates was performed at 16,000 *g* for 15 min, while the protein concentrations were determined using Bicinchoninic Acid (BCA) Kit (Thermo, United States). Cell lysates (20 μg) were separated by 10% SDS-PAGE and transferred to polyvinylidene difluoride membranes (Immobilon-P membranes; Millipore, Billerica, MA, United States). Membranes were blocked with blocking buffer (5% skim milk, 0.1% Tween-20 in PBS) for 1 h at room temperature. Following incubation with primary antibodies (1:1,000) overnight at 4°C, the membranes were incubated with horseradish peroxidase–conjugated secondary antibodies (Thermo Fisher Scientific, Inc. Waltham, MA, United States). Signals were detected using the ECL Plus Western Blotting Detection System (Amersham Biosciences, Piscataway, NJ, United States).

### Cell Counting Kit-8 Assay

HepG2 and L02 cells were plated in 96-well microtiter plates (Corning, United States) at a density of 3,000 cells per well. The cells were then treated with 2800Z, 40569Z, or in a combination with sorafenib. Cell Counting Kit-8 (CCK-8) assay (Dojin Laboratories, Japan) was performed to measure cell proliferation according to the manufacturer’s instructions. The absorbance was determined at 450 nm by a microplate reader.

### Colony Formation Assay

HepG2 cells were added to 24-well plates at a density of 5×10^3^ cells per well and then incubated for 5–7 additional days in the presence of 2800Z, 40569Z, or a combination with sorafenib. Cells were fixed by 10% formaldehyde and then stained with 0.1% crystal violet. Colony numbers were counted by at least five random fields.

### Immunoprecipitation

293T cells were seeded at 4 × 10^6^ cells/10 cm plate and transiently transfected with 4ug of Flag-SIRT7, Flag-SIRT6, Flag-SIRT1, or SIRT7 mutants. One day after transfection, cells were washed twice with PBS before harvest and were collected by RIPA buffer that contained 50 mM Tris, pH 7.5, 150 mM sodium chloride, 1% NP-40, 0.2% SDS, 0.5% sodium deoxycholate, 0.1 mM EDTA, and 1% protease and phosphatase inhibitors (Sigma-Aldrich). For each immunoprecipitation experiment, 400 μg cell extracts were subjected to immunoprecipitation with 50 μL anti-Flag M2 magnetic beads (Sigma-Aldrich).

### TUNEL Assay

Cell death was detected by TUNEL Assay Kit (C1090, Beyotime Institute of Biotechnology, Shanghai, China) according to the manufacturer’s instructions, and cell nuclei were labeled by 4′,6-diamidino-2-phenylindole (DAPI). Quantification of TUNEL staining was performed by examining at least five randomly selected fields.

### *In Vitro* Deacetylation Activity Assay

Deacetylation activity was assessed using a SIRT Deacetylase Activity Assay Kit (CS1040; Sigma-Aldrich) according to the manufacturer’s instructions. Equal amounts of purified SIRT proteins from transfection 293T were used for each experimental condition. SIRT deacetylase activity was measured using fluorescence intensity signals at 460 nm (excitation, 360 nm) as captured in a Synergy2 microplate fluorimeter (Bio Tek, Vermont, United States).

### Murine Xenograft Models

Male BALB/c-nu mice (4 weeks of age) were purchased from Gempharmatech (Nanjing, China). Mice were housed in a temperature-controlled, pathogen-free environment with 12-h light–dark cycles. All animal handling procedures were approved by the Institutional Animal Care and Use Committee at Hunan Normal University School of Medicine (Protocol 2020007-B). Mice were subcutaneously implanted in the right flank with 5 × 106 HepG2 cells. When tumors had grown to 40–60 mm^3^ in size, mice were randomly divided into control (100 µl 5% dextrose solution), sorafenib (3 mg/kg/day), 2800Z (4 mg/kg/day), and 40569Z (3 mg/kg/day) groups (n = 5 each group). Using a treatment of combinations, mice were randomized into control (100 µl 5% dextrose solution), sorafenib (2 mg/kg/day), 40569Z (1 mg/kg/day), 2800Z (2 mg/kg/day), and combination (2800Z 2 mg/kg/day and 40569Z 1 mg/kg/day combined with sorafenib 2 mg/kg/day, respectively). All treatments were administered intraperitoneally once in 2 days for 2 weeks. Tumor volumes and body weight were measured every 2 days, and tumor volume was calculated as follows: volume = 1/2 (length x width^2^). All mice were euthanized with sodium pentobarbital (Sigma Aldrich, United States) injection (150 mg/kg) according to AVMA Guidelines for the Euthanasia of Animals, and tumors were removed for further analysis.

### Histologic Analysis

Immunohistochemistry was performed as previously described (Li et al., 2018; Zhao et al., 2019). After deparaffinization and rehydration, antigen retrieval was achieved by heating in a pressure cooker for 5 min in 10 mM of sodium citrate (pH6). Peroxidase activity was blocked by incubation in 3% H_2_O_2_ for 10 min. Sections were rinsed three time in PBS/PBS-T (0.1% Tween-20) and incubated in Dako Protein Block (Dako, Agilent Technologies, Santa Clara, CA) for 10 min. After removal of blocking solution, slides were placed into a humidified chamber and incubated overnight at 4°C with primary antibodies in blocking buffer (4% normal goat serum in PBS). After washing, slides were covered with SignalStain Boost IHC Detection Reagent (Cell Signaling Technologies, Boston, MA) for 30 min at room temperature. After washing two times with PBS-T, the substrate-chromgen solution (VECTOR NovaRED, Substrate Kit, Vector Laboratories, Burlingame, CA) was applied, and the slides were incubated for 5–10 min and counterstained with hemtoxylin. Images were acquired using a Zeiss Axiolab 5 Digital Lab Microscope (Carl Zeiss AG, Jena, Germany).

### Statistical Analysis

Data are presented as mean ± sem. Statistical analysis was performed by using GraphPad Prism 6. Statistical significance between two groups was calculated by using one-way ANOVA and 2-tailed unpaired Student’s t-test, followed by Turkey’s test. Variance between groups met the assumptions of the appropriate test. Unless otherwise stated, a *p*-value of <0.05 was considered statistically significant.

## Results

### Protein Structure Prediction and Active Site Analysis of Human SIRT7

**FIGURE 1:**
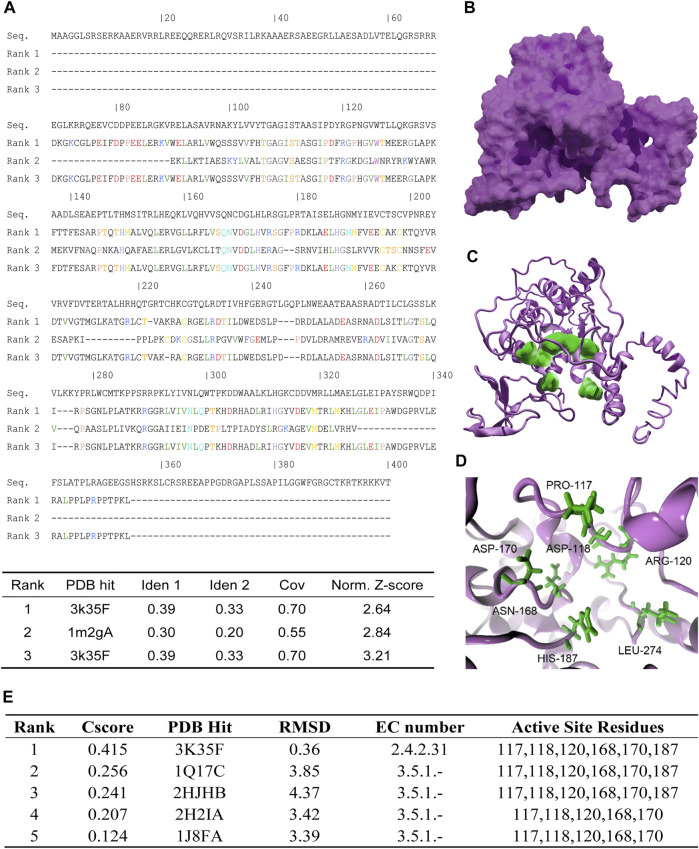
Protein structure prediction and analysis of human SIRT7. **(A)** Top 3 threading templates used by I-tasser. PDB hit indicates the PDB ID of the best aligned. Ident1 indicates percentage of sequence identity of the templates in the threading aligned region with the query sequence. Ident2 indicates the percentage sequence identity of the whole template chains with query sequence. Cov represents the coverage of the threading alignment and is equal to the number of aligned residues divided by the length of query protein. Norm. Z-score indicates the normalized Z-score of the threading alignments. **(B)** The threading result of human SIRT7: magenta surface represents SIRT7. **(C)** The active sites predict result of SIRT7, magenta cartoon represents SIRT7, and green surface represents the key residues. **(D)** Enlarged active sites of SIRT7: green sticks represent the key residues. **(E)** The predicted results of active site residues. Cscore indicates the confidence score of the prediction. Cscore ranges from 0 to 1, where a higher score indicates a more reliable prediction. PDB hit indicates the PDB ID of the aligned. RMSD indicates the RMSD between residues that were structurally aligned by TM-align. EC number represents the Enzyme Commission number which was a numerical classification scheme for enzymes. EC number 2.4.2.31 represented the protein was a NAD (+) protein-arginine ADP-ribosyltransferase. EC number 3.5.1 represented the protein was acting on carbon–nitrogen bonds other than peptide bonds in liner amides.

Due to the lack of SRIT7 crystal structure in a public database, we performed threading by using human SIRT7 full length sequence (accession number: NP_057622.1) and the structure of human SIRT6 (PDBID: 3K35) and human SIRT2 (PDBID:2H59) as templates. Top 3 threading templates used by *I-tasser* are shown in Figure 1A, and the obtained SIRT7 structure is shown in Figure 1B. We further analyzed the active sites of SIRT7 by *COFACTOR* and *COACH* based on the predicted structure. The results showed that the active residues of SIRT7 are PRO-117, ASP-118, ARG-120, ASN-168, ASP-170, and HIS-187 (colored in green in Figures 1C,D). To further validate those predicted results, we performed Ramachandran plot analysis and found that all the proline are in the allowable area (Supplementary Figure S1). We further performed Enzyme Commission (EC) number prediction to analyze enzymatic classification of the predicted structure. The results showed that the EC number of predicted structures was EC2.4.2.31, which refers to NAD (+)-protein-arginine ADP-ribosyltransferase and is consistent with intercellular functions of SIRT7 protein (Figure 1E).

### Structure-Based Virtual Screening for SIRT7 Inhibitors

**FIGURE 2:**
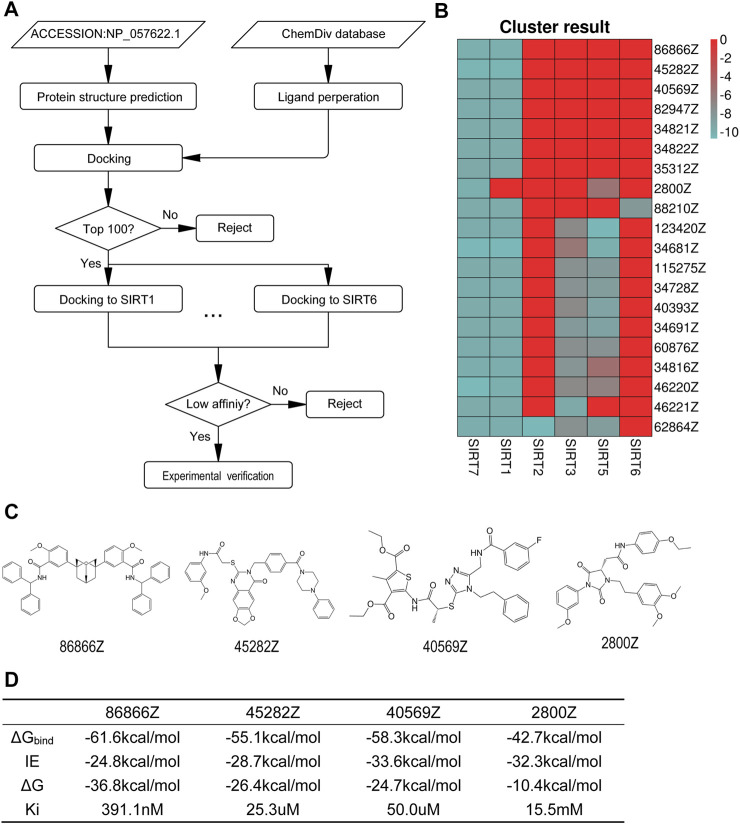
2800Z and 40569Z were the potential inhibitors to SIRT7. **(A)** The workflow of virtual screening of specific SIRT7 inhibitor. **(B)** The top 20 cluster results by *k-means* clustering algorithm in R-script. **(C)** Molecular formula of 86866Z (CID 5295932), 45282Z (CID 46370154), 40569Z (CID 99657111), and 2800Z (CID 97987669) which were selected for experiment verification. **(D)** The predicted results of binding free energy from molecular dynamics simulation (kcal/mol). ΔG_binding_ was the binding free energy calculated by MM-PBSA.py using the MM-GBSA method.

In order to screen specific SIRT7 inhibitors, we performed virtual screening using a molecular docking method by *Autodock vina*. The workflow for the structure prediction and virtual screening of this study is shown in Figure 2A. The docking grid was located on the predicted active sites mentioned above, and all possible active residues were also included. In order to develop specific SIRT7 inhibitor, we tested 939319 structurally diverse compounds from Chemdiv database as ligands, and the top 100 molecules that showed high affinity to SIRT7 were selected for further docking to other family members of SIRT proteins except SIRT4 due to the lack of the crystal structure. All the results were integrated into vectors based on the molecular name, and the contents of vectors were the affinities to SIRT proteins. Vectors were clustered by *k-means* algorithm, and the top 20 compounds with high selectivity to SIRT7 but low affinities to other SIRT proteins are shown in Figure 2B. The top eight specific SIRT7 inhibitors were 86866Z, 45282Z, 40569Z, 82947Z, 34821Z, 34822Z, 35312Z, and 2800Z. However, 82947Z, 34821Z, 34822Z, and 35312Z were not available. Thus, four molecules, including 86866Z, 45282Z, 40569Z, and 2800Z (Figure 2C), were used for further predictions of their dissociation constants with the receptor from molecular dynamics simulations (Figure 2D). We obtained compounds 86866Z, 45282Z, 40569Z, and 2800Z from Chemdiv and tested their activities *in vitro*. Compound 45282 showed poor solubility, and our initial experimental results indicated that compounds 40569Z and 2800Z showed the best water solubilities and drug abilities (Supplementary Figure S2); we thus chose these two compounds for further investigations.

### Molecular Dynamics Simulation of Compounds and SIRT7 Complex

**FIGURE 3:**
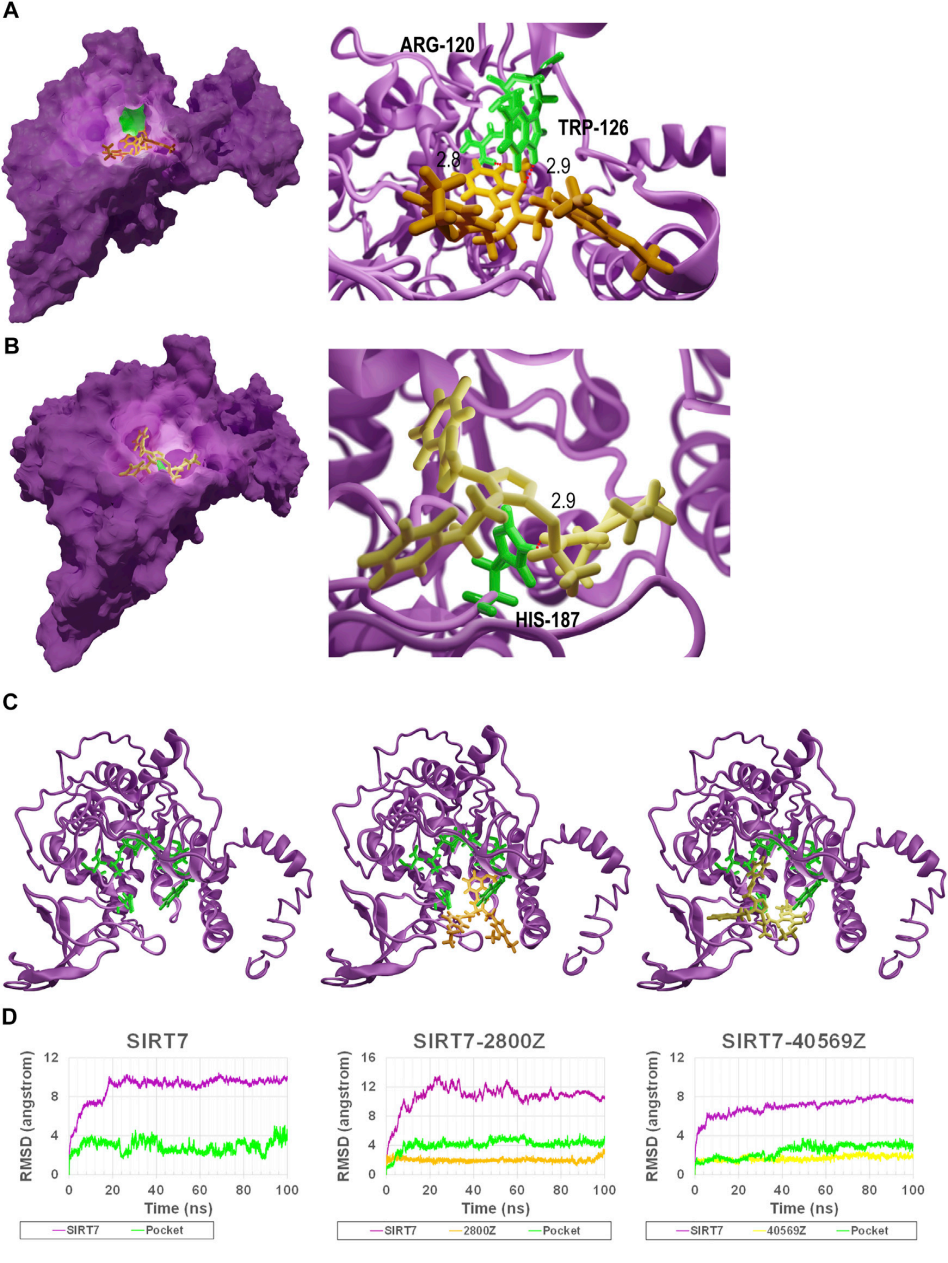
Binding model of 40569Z-SIRT7 and 2800Z-SIRT7 complexes. **(A,B)** The SIRT7–40569Z and SIRT7–2800Z complex from docking: the magenta surface on the left represents SIRT7, the green surface on the left represents the key residues in SIRT7–compound interactions, and the orange and yellow sticks represent 2800Z and 40569Z, respectively. The red dash line on the right represents the hydrogen bond. Numbers indicate the distance between compounds and residues. **(C,D)** RMSD for wild-type SIRT7, SIRT7–2800Z complex, and SIRT7–40569Z complex in 100 ns MD simulations. Magenta line in chart and magenta cartoon(C) indicates the SIRT7, green line in chart and green sticks (C) represent the predicted interaction pocket. Yellow sticks represent 40569Z, and orange sticks represent 2800Z.

We investigated the interactions of SIRT7 protein with compounds 2800Z and 40569Z based on their docking structures. We found that residues ARG-120 and TRP-126 (colored in green in Figure 3A) were critical for SIRT7–2800Z to form hydrogen bonds, while residue HIS-187 (colored in green in Figure 3B) was critical for SIRT7–40569Z interactions. We also found that the sub-binding pockets of 2800Z (colored in orange) and 40569Z (colored in yellow) were different (Figure 3C). We further evaluated the interactions between these compounds and SIRT7 through molecular dynamics simulations*.* The SIRT7–2800Z complex and SIRT7–40569Z complex were simulated for 100 ns. The root-mean-square deviations (RMSDs) of all the heavy atoms were calculated with reference to their initial conformations. The results (Figure 3D) indicated that after binding to compounds 40569Z and 2800Z, the structures of SIRT7 changed in the first 10ns when compared with its initial conformations (Figure 3C), which were caused by the first 50 residues in the N-terminal. Notably, even the first 50 residues in the N-terminal were in an unstable state, but the binding pockets (green lines in chart and green cartoon in Figure 3C) were not affected.

### Specificity of Compounds 2800Z and 40569Z in Inhibiting SRIT7 Deacetylation Activity

**FIGURE 4:**
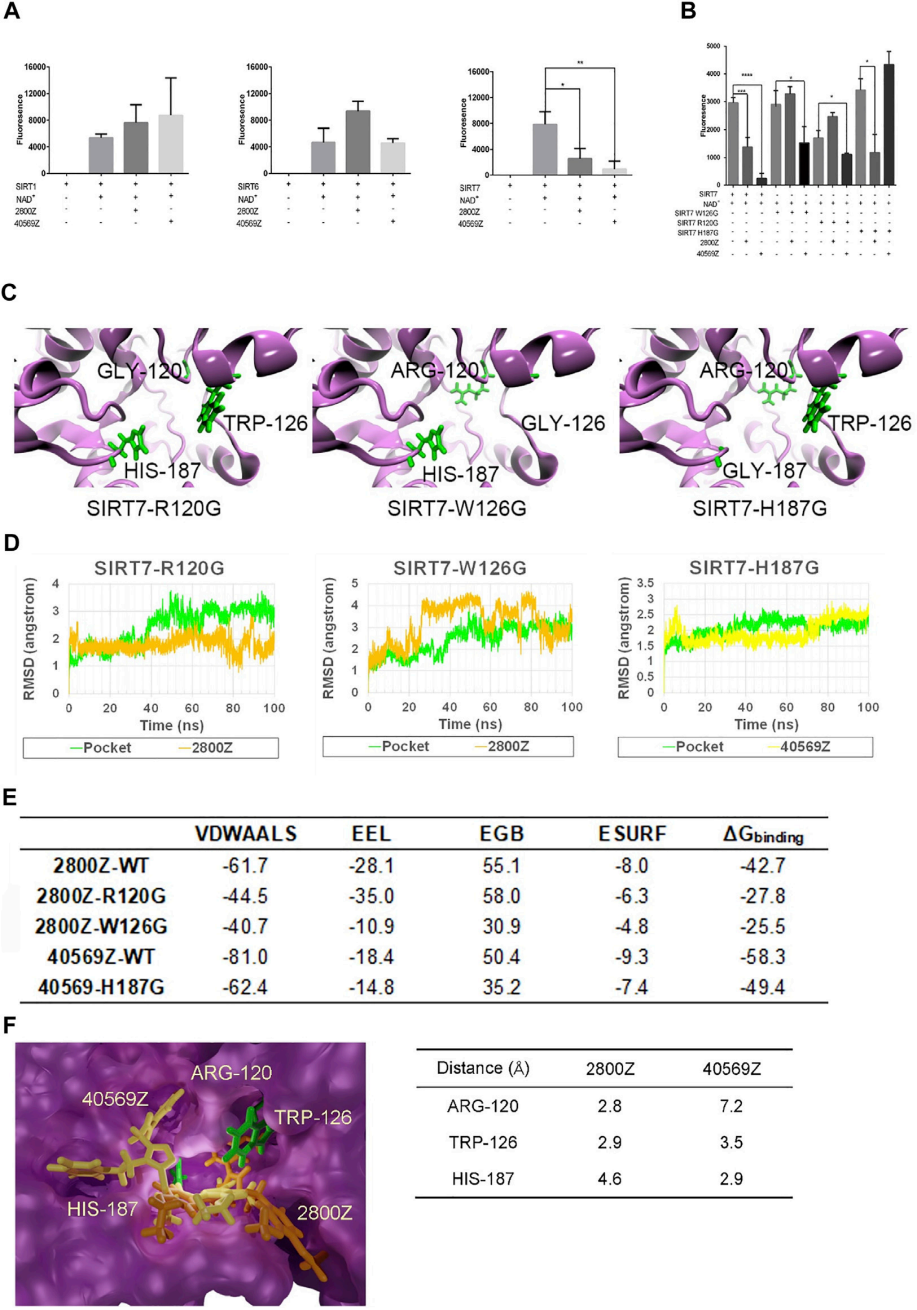
2800Z and 40569Z are the specific inhibitors of SIRT7. **(A)** Flag tagged SIRT1, SIRT6, and SIRT7 were transfected with 293T cells for 24 h, and the proteins were purified by immunoprecipitation (IP), deacetylation activity of SIRT1, SIRT6, and SIRT7 was measured in the presence or absence of compounds 2800Z and 40569Z, respectively. *p < 0.05, **p < 0.01 vs SIRT7+NAD^+^, one-way ANOVA. Graphs show mean ± SEM of at least three independent experiments. **(B)** Flag tagged SIRT7, SIRT7 R120G, SIRT7 W126G, and SIRT7 H187G were transfected with 293T cells for 24 h, and the proteins were purified by IP, deacetylation activity was measured in the presence or absence of compounds 2800Z and 40569Z. *p < 0.05***p < 0.001, ****p < 0.0001, one-way ANOVA. Graphs show mean ± SEM of at least three independent experiments. **(C)** Schematic structure of SIRT7 mutants: green sticks represent the key residues. **(D)** RMSD for three types of mutated complex. **(E)** Binding free energies of 2800Z and 40569Z to SIRT7 and mutants (kcal/mol). **(F)** Diagram of the different interactions of 2800Z and 40569Z to SIRT7: green sticks indicate the key residues in protein–ligand interaction, yellow sticks represent 40569Z, and orange sticks represent 2800Z.

To confirm the specificity of compounds 2800Z and 40569Z, we performed *in vitro* deacetylation assay. Flag-tagged SIRT1, SIRT6, and SIRT7 were purified by immunoprecipitation (IP) and incubated with SIRT substrates in the presence or absence of these compounds. As shown in Figure 4A, in the absence of NAD+, SIRT proteins showed no deacetylation activities. We found that both 2800Z and 40569Z significantly inhibited SIRT7 deacetylation activity but had nearly no effects on SIRT1 and SIRT6 activities. To further investigate potential mechanisms underlying inhibition of these two compounds on SIRT7-dependent deacetylation, we generated three SIRT7 mutants in which the main predicted interaction sites for these two compounds as shown in Figures 2C,D including TRP-126, ARG-120, and HIS-187 were replaced with glycine (Figure 4B). We found that replacement of ARG-120 to glycine (SIRT7 R120G) slightly decreased SIRT7 deacetylation activity at the basal level, while mutation with the other two sites had no effects. In case of 2800Z, replacing either TRP-126 (SIRT7 W126G) or ARG-120 to glycine completely abolished the inhibitory effects of 2800Z when compared to wild-type (WT) SIRT7. In contrast, 2800Z significantly suppressed deacetylation activities of SIRT7 H187G which replaced HIS-187 to glycine. On the other hand, mutant of SIRT7 H187G completely abolished inhibitory effects of 40569Z when compared to WT SIRT7 but had nearly no effects on SIRT7 R120G and SIRT7 W126G (Figure 4B). These data suggested that TRP-126 and ARG-120 are essential for 2800Z, while HIS-187 is important for 40569Z-mediated SIRT7 inhibition. To further reveal how mutation of these residues impacted SIRT7 compound interactions, we first performed MD simulations by using structures of SIRT7 mutants (Figure 4C, green sticks represent the key residues). RMSDs of all the heavy atoms were also calculated with reference to their initial conformations (Figure 4D). The results of RMSD indicated that compound 2800Z in the SIRT7 W126G–2800Z complex was unstable when compared with SIRT7 R120G–2800Z and WT SIRT7–2800Z. Then, the binding free energies of 2800Z to SIRT7 R120G and SIRT7 W126G, and 40569Z to SIRT7 H187G were calculated by the MM-GBSA method (Figure 4E). The binding free energies of SIRT7 R120G–2800Z (-3.1 kcal/mol), SIRT7 W126G–2800Z (0.1 kcal/mol), and SIRT7 H187G–40569Z (-17.9 kcal/mol) were all increased compared to the WT SIRT7–compound complexes (-10.4 kcal/mol for WT SIRT7–2800Z and -24.7 kcal/mol for WT SIRT7–40569Z), suggesting the impairment of SIRT7–compound interaction when mutating those sites. Especially, the binding free energy of the SIRT7-W126G–2800Z complex increased to 0.1 kcal/mol, indicating the pocket was not specified for 2800Z. Mutation of TRP-126 directed causes the unstable of SIRT7–2800Z interaction also suggested TRP-126 was the key residue in the interaction between 2800Z and SIRT7 (Figure 4E). To investigate potential mechanisms underlying residue preferences of SIRT7–compound interactions, we measured the distance of the two closest atoms between each compound and residues TRP-126, ARG-120, and HIS-187 of SIRT7 (Figure 4F). Consistent with observations in Figure 4E, the results indicated that interactions of 40569Z and SIRT7 rely on HIS-187 (2.9 Å), while 2800Z and SIRT7 require ARG-120 (2.8 Å) and TRP-126 (2.9 Å), respectively.

### Anticancer Effects of 2800Z and 40569Z *In Vitro*

**FIGURE 5:**
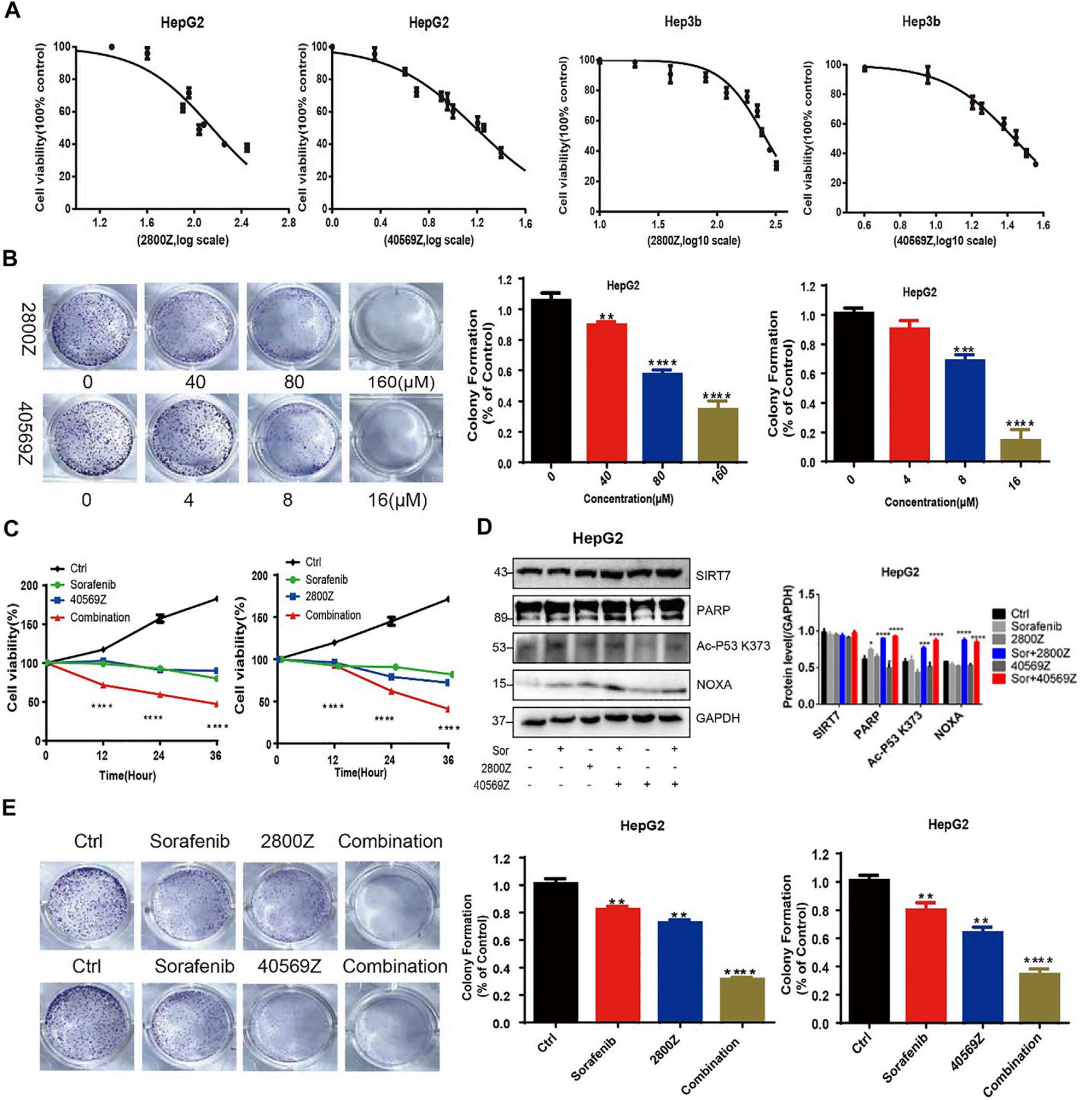
*In vitro* assessment of anticancer effects of 2800Z and 40569Z. **(A)** HepG2 cells were treated with a range of concentration of 2800Z or 40569Z, and cell viability was assessed 24 h after treatment by using the CCK8 assay. **(B)** Colony formation assay of HepG2 cells was untreated or treated with 2800Z or 40569Z in a dose-dependent fashion for 5 days. Graphs show mean ± SEM of at least three independent experiments. **p < 0.01, ***p < 0.001, ****p < 0.0001, one-way ANOVA. (C–D) HepG2 cells were treated with sorafenib (4 μM) in the absence or presence of 2800Z (60 μM) or 40569Z (6 μM), and cell viability was assessed at various time points as indicated by using the CCK8 assay **(C)**, and protein levels of SIRT7, PARP, acetyl-p53 K373, and NOXA were evaluated by WB **(D)**. Quantification of WB was determined by densitometry. Graphs show mean ± SEM of at least three independent experiments. *p < 0.05, ***p < 0.001, ****p < 0.0001, one-way ANOVA. **(E)** Colony formation assay of HepG2 cells was untreated or treated sorafenib (4 μM) in the absence or presence of 2800Z (60 μM) or 40569Z (6 μM) for 5 days **p < 0.01, ****p < 0.0001. All graphs show mean ± SEM of at least three independent experiments.

We have previously reported inactive SIRT7 sensitizes HCC to doxorubicin, which prompts us to further assess anticancer effects of 2800Z and 40569Z. We first measured IC_50_ values of these two compounds by using HepG2 and normal hepatocyte cell line L02 cells. As shown in Figure 5A, IC_50_ values of 2800Z and 40569Z were 134 μM and 13 μM in HepG2, and 165 μM and 96 μM in L02 cells, respectively. Both 2800Z and 40569Z significantly inhibited colony formations of HepG2 cells in a dose-dependent manner (Figure 5B). To further assess whether these two compounds could enhance cytotoxicity of sorafenib, we treated HepG2 cells with sorafenib (4 μM) in the absence or presence of 2800Z (60 μM) or 40569Z (6 μM) and measured cell viability (Figure 5C). Sorafenib, 2800Z, and 40569Z slightly decreased cell viability, but both 2800Z and 40569Z were able to increase sorafenib cytotoxicity in HepG2 cells (Figure 5C). Synergy between these compounds and sorafenib were assessed based on synergy finder as previously described (Ianevski et al., 2020). The synergy scores of compounds 2800Z, 40569Z, and sorafenib were 4.793 and 6.168, respectively (Supplementary Figure S3), which indicated the interaction between compounds and sorafenib was likely to be additive. We have previously demonstrated that SIRT7 regulates doxorubicin chemosensitivity by inducing p53 K320 and K372 deacetylation and suppressing p53-dependent NOXA expression (Zhao et al., 2019). We thus evaluated whether these two compounds increase sorafenib cytotoxicity through the same mechanisms. Unlike doxorubicin, sorafenib did not change the expression level of SIRT7 but slightly increased NOXA expression. Combining sorafenib with either 2800Z or 40569Z significantly increased the ability of sorafenib-induced acetylation levels of p53 K373 and NOXA, cleaved PARP expression (Figure 5D), and inhibited colony formation of HepG2 cells (Figure 5E). These data clearly indicated compounds 2800Z and 40569Z increase sorafenib cytotoxicity by targeting SIRT7/p53 pathway *in vitro*.

### 2800Z and 40569Z Sensitize Xenograft HCC Tumor to Sorafenib *In Vivo*

**FIGURE 6:**
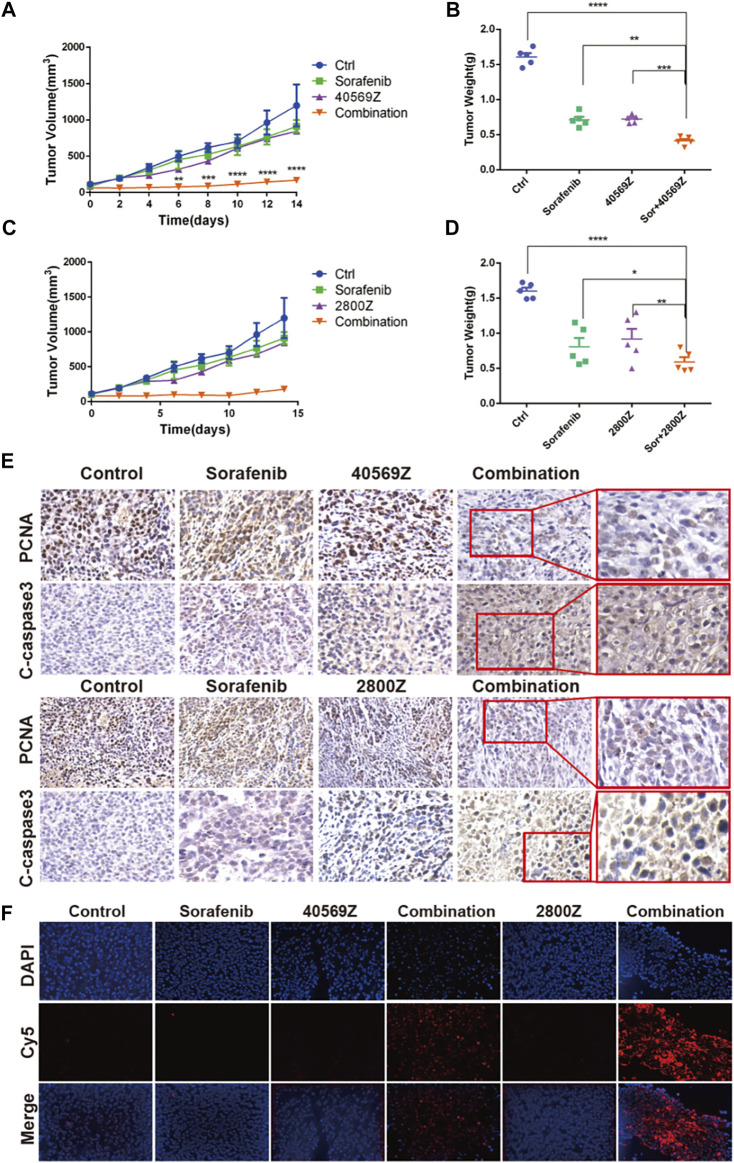
*In vivo* assessment of anticancer effects of 2800Z and 40569Z. **(A)** Tumor growth curves of tumor bearing nude mice that received vehicle, 2800Z (4 mg/kg), 40569Z (3 mg/kg) or sorafenib (3 mg/kg) treatment for 2 weeks. Graphs show mean ± SEM. ****p < 0.0001 VS sorafenib, one-way ANOVA. **(B)** Tumor weight after treatment as in A. Graphs show mean ± SEM. ***p < 0.001, ****p < 0.0001, VS control, one-way ANOVA. **(C)** Tumor growth curves of tumor that received vehicle, 2800Z (2 mg/kg), 40569Z (1 mg/kg), sorafenib (2 mg/kg), or combination (2800Z or 40569Z and sorafenib) treatment for 2 weeks. Graphs show mean ± SEM. ****p < 0.0001, VS control, one-way ANOVA. **(D)** Differences in tumor weight. Graphs show mean ± SEM. *p < 0.05, **p < 0.01, ****p < 0.0001. All graphs show mean ± SEM of at least three independent experiments. Control, one-way ANOVA. **(E, F)** Representative IHC staining of PCNA and cleaved-caspase3 (C-caspase) **(E)** and TUNEL staining **(F)** in tumors as in B and D.

We further evaluated anticancer effects of 2800Z and 40569Z *in vivo* by using a xenograft mouse model. We detected the purities of compounds by HPLC before *in vivo* experiments, and the results indicated that purities of both compounds were higher than 95% (Supplementary Figure S4). We found that 2800Z (2 mg/kg), 40569Z (1 mg/kg), and sorafenib (2 mg/kg) treatment alone showed minor effects on tumor growth. Combining sorafenib with either compound significantly lowered the tumor growth rate (Figure 6A,C) and decreased tumor weight when compared with using them alone (Figure 6B,D). We further assessed tumor proliferation and apoptosis by using IHC staining and TUNEL assay (Figures 6E,F). The results indicated that combining sorafenib with either compound significantly suppressed tumor proliferation and increased apoptosis evidenced by PCNA and cleaved-caspase (c-caspase 3) staining (Figure 6E). Similar results were observed in which more increased TUNEL-positive cells were present in tumors of combination groups (Figure 6F).

## Discussion

In the present study, we used structure-based virtual screening to develop SIRT7 inhibitor and validated their functions both *in vitro* and *in vivo*. Due to the lack of the crystal structure of SIRT7, we predicted the structure of SIRT7 by using the fold recognition (or threading) method (Yang et al., 2015; Yang and Zhang, 2015). Then, 939319 structurally diverse compounds from the Chemdiv database were docked to the predicted structure of SIRT7, and the top 100 candidate compounds that showed high-affinity toward SIRT7 were selected to further test their affinity toward other SIRT proteins. The top 20 compounds that have high affinities toward SIRT7 but low affinity for other SIRT proteins were selected as candidates for SIRT7 inhibitors. We further validated specificity and biological activities of candidate compounds. As a result, our leading compounds, 2800Z and 40569Z, showed strong interactions with SIRT7 protein, specifically inhibited SIRT7 deacetylation activity *in vitro* and *in vivo* data indicated these compounds induced apoptosis, and increased chemosensitivity to Sorafenib in human liver cancer. In line with many other observations (Malik et al., 2015; Kim et al., 2019), our data further proved the importance of SIRT7 in human cancer which may serve as an attractive druggable target.

We analyzed the active sites of SIRT7 by *COFACTOR* (Roy et al., 2012; Zhang et al., 2017) and *COACH* (Yang et al., 2013a; Yang et al., 2013b) based on the predicted structure, and the results showed that the active residues of SIRT7 are PRO-117, ASP-118, ARG-120, ASN-168, ASP-170, and HIS-187. Whether these residues regulate SIRT7 activities remain unclear and are worth further investigation. Based on the docking results, we successfully identified three residues of ARG-120 TRP-126 and HIS-187 that might be the key residues responsible for SIRT7 candidate compound interactions. Single mutations of the aforementioned three sites itself had nearly no effects on SRIT7 deacetylation activities but significantly abolished inhibitory effects of compounds 2800Z and 40569Z on SIRT7 activities. MD simulations and binding free energy calculations further uncovered that all three sites were essential for SIRT7 compounds interaction. Our data thus provided crucial sites for developing SIRT7 inhibitors, and the crystal structure of SIRT7 is also desirable for a more accurate drug design.

SIRT7 is a NAD^+^-dependent nuclear deacetylase, and mounting evidences support the critical roles of SIRT7 in multiple processes regulating physiological and pathological states by targeting a wide spectrum of substrates including p53 (Vakhrusheva et al., 2008), GABP-β (Ryu et al., 2014), FOXO3 (Li et al., 2017), and U3-55k (Chen et al., 2016) for deacetylation. In particular, SIRT7 is also implicated in human cancer by modulating key processes linked to cell fate determination and oncogenesis such as genome stability (Song et al., 2017), DNA damage repair (Vazquez et al., 2016; Zhang et al., 2016), and apoptosis (Li et al., 2019). Altered SIRT7 expression is frequently observed in many human cancers, and high SIRT7 is associated with aggressive cancer phenotype, distance metastasis, and poor survival (Barber et al., 2012; Yu et al., 2014; Zhao et al., 2019). Emerging evidence point toward SIRT7 as a therapeutic target for human cancer management because inactivate SIRT7 results in impairment of cancer transformation, increases chemosensitivity, and reverses metastatic phenotypes in both epithelial and mesenchymal tumors (Malik et al., 2015; Monteiro-Reis et al., 2020; Zhao et al., 2020). By using compound screening, Kim et al. (2019) reported that compounds inhibit SIRT7 enzyme activity and suppress uterine sarcoma growth *in vivo*. Together with previous work, our data clearly support that targeting SIRT7 potentiates the mechanism-based translational therapeutic strategy for liver cancer management. Further works focus on optimization of potency and selectivity; anticancer effects on other types of cancer and pharmacological implications of these compounds would be of interest.

Multiple lines of evidence indicate that inactive SIRT7 suppresses tumor growth, and our data also show that compounds 2800Z and 40569Z specifically inhibit SIRT7 enzyme activity and suppress tumor growth. Whether SIRT7 is responsible for this inhibition is currently under investigation in our lab. Nevertheless, we previously reported that inactive SIRT7 sensitizes human liver cancer to doxorubicin *via* deacetylation p53 and upregulates NOXA expression. In the absence of p53, SIRT7 no longer suppresses doxorubicin or induces apoptosis (Zhao et al., 2019). In this study, we do observe similar results that inhibit SIRT7 with compounds 2800Z and 40569Z increase Sorafenib induced p53 acetylation, NOXA expression, and cell apoptosis both *in vitro* and *in vivo*. Our data thus suggest these compounds increase liver cancer sensitivity to sorafenib mainly through SIRT7/p53/NOXA axis.

## Conclusion

Our findings demonstrated targeting SIRT7 may offer novel therapeutic options for cancer management, and the value of compounds 2800Z and 40569Z as chemical probes for the study of SIRT7 biological functions as well as starting leads for the development of new therapeutic options against liver cancer.

## Data Availability

The original contributions presented in the study are included in the article/Supplementary Material; further inquiries can be directed to the corresponding authors.

## Abbreviation

TKI, tyrosine kinase inhibitor; OS, overall survival; PFS, progression-free survival; HDAC III, class III histone deacetylases; Sir2, silent information regulator 2; IP, immunoprecipitation; RMSD, root-mean-square deviations; DMEM, Dulbecco’s modified Eagle’s medium; FBS, fetal bull serum; PBS, phosphate-buffered saline; CCK-8, cell counting kit-8; H&E, hematoxylin and eosin; TUNEL, terminal deoxynucleotidyl transferase–mediated dUTP-biotin nick end-labeling.

## References

[B1] Barber M. F. Michishita-Kioi E. Xi Y. Tasselli L. Kioi M. Moqtaderi Z. (2012). SIRT7 Links H3K18 Deacetylation to Maintenance of Oncogenic Transformation. Nature 487 (7405), 114–118. 10.1038/nature11043 22722849 PMC3412143

[B2] Bruix J. Chan S. L. Galle P. R. Rimassa L. Sangro B. (2021). Systemic Treatment of Hepatocellular Carcinoma: An EASL Position Paper. J. Hepatol. 75, 960–974. 10.1016/j.jhep.2021.07.004 34256065

[B3] Case D. A. Belfon K. Ben-Shalom I. Y. Brozell S. R. Cerutti D. S. Cheatham T. E. (2021). AMBER 2020. San Francisco: University of California.

[B4] Chen S. Blank M. F. Iyer A. Huang B. Wang L. Grummt I. (2016). SIRT7-dependent Deacetylation of the U3-55k Protein Controls Pre-rRNA Processing. Nat. Commun. 7, 10734. 10.1038/ncomms10734 26867678 PMC4754350

[B5] Chen S. Seiler J. Santiago-Reichelt M. Felbel K. Grummt I. Voit R. (2013). Repression of RNA Polymerase I upon Stress Is Caused by Inhibition of RNA-dependent Deacetylation of PAF53 by SIRT7. Mol. Cel 52 (3), 303–313. 10.1016/j.molcel.2013.10.010 24207024

[B6] Cheng Z. Li X. Hou S. Wu Y. Sun Y. Liu B. (2019). K-Ras-ERK1/2 Accelerates Lung Cancer Cell Development via Mediating H3K18ac through the MDM2-GCN5-SIRT7 axis. Pharm. Biol. 57 (1), 701–709. 10.1080/13880209.2019.1672756 31613681 PMC6807650

[B7] Dyhl-Polk A. Mikkelsen M. K. Ladekarl M. Nielsen D. L. (2021). Clinical Trials of Immune Checkpoint Inhibitors in Hepatocellular Carcinoma. Jcm 10 (12), 2662. 10.3390/jcm10122662 34208788 PMC8234948

[B8] European Association for the Study of the Liver (2018). Electronic address, e.e.e., and European Association for the Study of the, LEASL Clinical Practice Guidelines: Management of hepatocellular carcinoma. J. Hepatol. 69 (1), 182–236. 10.1016/j.jhep.2018.03.019 29628281

[B9] Heimbach J. K. Kulik L. M. Finn R. S. Sirlin C. B. Abecassis M. M. Roberts L. R. (2018). AASLD Guidelines for the Treatment of Hepatocellular Carcinoma. Hepatology 67 (1), 358–380. 10.1002/hep.29086 28130846

[B10] Ianevski A. Giri A. K. Aittokallio T. (2020). SynergyFinder 2.0: Visual Analytics of Multi-Drug Combination Synergies. Nucleic Acids Res. 48 (W1), W488–w493. 10.1093/nar/gkaa216 32246720 PMC7319457

[B11] Jakalian A. Jack D. B. Bayly C. I. (2000). Fast, Efficient Generation of High-Quality Atomic Charges. AM1-BCC Model: I. Method. J. Comput. Chem. 21 (2), 132–1246. 10.1002/jcc.10128 12395429

[B12] Jorgensen W. L. Chandrasekhar J. Madura J. D. Impey R. W. Klein M. L. (1983). Comparison of Simple Potential Functions for Simulating Liquid Water. J. Chem. Phys. 79 (2), 926–935. 10.1063/1.445869

[B13] Kim J.-H. Kim D. Cho S. J. Jung K.-Y. Kim J.-H. Lee J. M. (2019). Identification of a Novel SIRT7 Inhibitor as Anticancer Drug Candidate. Biochem. Biophysical Res. Commun. 508 (2), 451–457. 10.1016/j.bbrc.2018.11.120 30503501

[B14] Kim J. K. Noh J. H. Jung K. H. Eun J. W. Bae H. J. Kim M. G. (2013). Sirtuin7 Oncogenic Potential in Human Hepatocellular Carcinoma and its Regulation by the Tumor Suppressors MiR-125a-5p and MiR-125b. Hepatology 57 (3), 1055–1067. 10.1002/hep.26101 23079745

[B15] Li H. Tian Z. Qu Y. Yang Q. Guan H. Shi B. (2019). SIRT7 Promotes Thyroid Tumorigenesis through Phosphorylation and Activation of Akt and p70S6K1 via DBC1/SIRT1 axis. Oncogene 38 (3), 345–359. 10.1038/s41388-018-0434-6 30093629

[B16] Li Z. Bridges B. Olson J. Weinman S. A. (2017). The Interaction between Acetylation and Serine-574 Phosphorylation Regulates the Apoptotic Function of FOXO3. Oncogene 36 (13), 1887–1898. 10.1038/onc.2016.359 27669435 PMC5366279

[B17] Li Z. Zhao J. Zhang S. Weinman S. A. (2018). FOXO3-dependent Apoptosis Limits Alcohol-Induced Liver Inflammation by Promoting Infiltrating Macrophage Differentiation. Cel Death Discov. 4, 16. 10.1038/s41420-017-0020-7 PMC5841311 29531813

[B18] Llovet J. M. Villanueva A. Lachenmayer A. Finn R. S. (2015). Advances in Targeted Therapies for Hepatocellular Carcinoma in the Genomic Era. Nat. Rev. Clin. Oncol. 12 (7), 408–424. 10.1038/nrclinonc.2015.103 26054909

[B19] Malik S. Villanova L. Tanaka S. Aonuma M. Roy N. Berber E. (2015). SIRT7 Inactivation Reverses Metastatic Phenotypes in Epithelial and Mesenchymal Tumors. Sci. Rep. 5, 9841. 10.1038/srep09841 25923013 PMC4413894

[B20] Miller B. R. 3rd McGee T. D. Jr. Swails J. M. Homeyer N. Gohlke H. Roitberg A. E. (2012). MMPBSA.py: An Efficient Program for End-State Free Energy Calculations. J. Chem. Theor. Comput. 8 (9), 3314–3321. 10.1021/ct300418h 26605738

[B21] Miller K. D. Fidler‐Benaoudia M. Keegan T. H. Hipp H. S. Jemal A. Siegel R. L. (2020). Cancer Statistics for Adolescents and Young Adults, 2020. CA A. Cancer J. Clin. 70 (6), 443–459. 10.3322/caac.21637 32940362

[B22] Mlynarsky L. Menachem Y. Shibolet O. (2015). Treatment of Hepatocellular Carcinoma: Steps Forward but Still a Long Way to Go. Wjh 7 (3), 566–574. 10.4254/wjh.v7.i3.566 25848480 PMC4381179

[B23] Monteiro-Reis S. Lameirinhas A. Miranda-Gonçalves V. Felizardo D. Dias P. C. Oliveira J. (2020). Sirtuins' Deregulation in Bladder Cancer: SIRT7 Is Implicated in Tumor Progression through Epithelial to Mesenchymal Transition Promotion. Cancers 12 (5), 1066. 10.3390/cancers12051066 PMC7281198 32344886

[B24] Perera S. Kelly D. O’Kane G. M. (2020). Non-immunotherapy Options for the First-Line Management of Hepatocellular Carcinoma: Exploring the Evolving Role of Sorafenib and Lenvatinib in Advanced Disease. Curr. Oncol. 27 (Suppl. 3), 165–172. 10.3747/co.27.7159 33343210 PMC7739521

[B25] Ponder J. W. Case D. A. (2003). Force fields for Protein Simulations. Adv. Protein Chem. 66 (1), 27–85. 10.1016/s0065-3233(03)66002-x 14631816

[B26] Roy A. Kucukural A. Zhang Y. (2010). I-TASSER: a Unified Platform for Automated Protein Structure and Function Prediction. Nat. Protoc. 5 (4), 725–738. 10.1038/nprot.2010.5 20360767 PMC2849174

[B27] Roy A. Yang J. Zhang Y. (2012). COFACTOR: an Accurate Comparative Algorithm for Structure-Based Protein Function Annotation. Nucleic Acids Res. 40, W471–W477. 10.1093/nar/gks372 22570420 PMC3394312

[B28] Ryu D. Jo Y. S. Lo Sasso G. Stein S. Zhang H. Perino A. (2014). A SIRT7-dependent Acetylation Switch of GABPβ1 Controls Mitochondrial Function. Cel Metab. 20 (5), 856–869. 10.1016/j.cmet.2014.08.001 25200183

[B29] Sangro B. Carpanese L. Cianni R. Gasparini D. Golfieri R. Ezziddin S. (2012). Assessment of Radioembolization Among Patients Who Met the Inclusion Criteria for Sorafenib Hepatocellular Carcinoma Assessment Randomized Protocol (SHARP). J. Clin. Oncol. 30 (15), 1. 10.1200/jco.2012.30.15_suppl.e14654 22105825

[B30] Siegel R. L. Miller K. D. Jemal A. (2020). Cancer Statistics, 2020. CA A. Cancer J. Clin. 70 (1), 7–30. 10.3322/caac.21590 31912902

[B31] Song C. Hotz-Wagenblatt A. Voit R. Grummt I. (2017). SIRT7 and the DEAD-Box Helicase DDX21 Cooperate to Resolve Genomic R Loops and Safeguard Genome Stability. Genes Dev. 31 (13), 1370–1381. 10.1101/gad.300624.117 28790157 PMC5580657

[B32] Sung H. Ferlay J. Siegel R. L. Laversanne M. Soerjomataram I. Jemal A. (2021). Global Cancer Statistics 2020: GLOBOCAN Estimates of Incidence and Mortality Worldwide for 36 Cancers in 185 Countries. CA A. Cancer J. Clin. 71 (3), 209–249. 10.3322/caac.21660 33538338

[B33] Trott O. Olson A. J. (2009). AutoDock Vina: Improving the Speed and Accuracy of Docking with a New Scoring Function, Efficient Optimization, and Multithreading. J. Comput. Chem. 31 (2), NA. 10.1002/jcc.21334 PMC3041641 19499576

[B34] Vakhrusheva O. Smolka C. Gajawada P. Kostin S. Boettger T. Kubin T. (2008). Sirt7 Increases Stress Resistance of Cardiomyocytes and Prevents Apoptosis and Inflammatory Cardiomyopathy in Mice. Circ. Res. 102 (6), 703–710. 10.1161/CIRCRESAHA.107.164558 18239138

[B35] Vazquez B. N. Thackray J. K. Simonet N. G. Kane‐Goldsmith N. Martinez‐Redondo P. Nguyen T. (2016). SIRT 7 Promotes Genome Integrity and Modulates Non‐homologous End Joining DNA Repair. EMBO J. 35 (14), 1488–1503. 10.15252/embj.201593499 27225932 PMC4884211

[B36] Yang J. Roy A. Zhang Y. (2013a). BioLiP: a Semi-manually Curated Database for Biologically Relevant Ligand-Protein Interactions. Nucleic Acids Res. 41, D1096–D1103. 10.1093/nar/gks966 23087378 PMC3531193

[B37] Yang J. Roy A. Zhang Y. (2013b). Protein-ligand Binding Site Recognition Using Complementary Binding-specific Substructure Comparison and Sequence Profile Alignment. Bioinformatics 29 (20), 2588–2595. 10.1093/bioinformatics/btt447 23975762 PMC3789548

[B38] Yang J. Yan R. Roy A. Xu D. Poisson J. Zhang Y. (2015). The I-TASSER Suite: Protein Structure and Function Prediction. Nat. Methods 12 (1), 7–8. 10.1038/nmeth.3213 25549265 PMC4428668

[B39] Yang J. Zhang Y. (2015). I-TASSER Server: New Development for Protein Structure and Function Predictions. Nucleic Acids Res. 43 (W1), W174–W181. 10.1093/nar/gkv342 25883148 PMC4489253

[B40] Yu H. Ye W. Wu J. Meng X. Liu R.-y. Ying X. (2014). Overexpression of Sirt7 Exhibits Oncogenic Property and Serves as a Prognostic Factor in Colorectal Cancer. Clin. Cancer Res. 20 (13), 3434–3445. 10.1158/1078-0432.Ccr-13-2952 24771643

[B41] Zhang C. Freddolino P. L. Zhang Y. (2017). COFACTOR: Improved Protein Function Prediction by Combining Structure, Sequence and Protein-Protein Interaction Information. Nucleic Acids Res. 45 (W1), W291–w299. 10.1093/nar/gkx366 28472402 PMC5793808

[B42] Zhang P.-Y. Li G. Deng Z.-J. Liu L.-Y. Chen L. Tang J.-Z. (2016). Dicer Interacts with SIRT7 and Regulates H3K18 Deacetylation in Response to DNA Damaging Agents. Nucleic Acids Res. 44 (8), 3629–3642. 10.1093/nar/gkv1504 26704979 PMC4856966

[B43] Zhao J. Wozniak A. Adams A. Cox J. Vittal A. Voss J. (2019). SIRT7 Regulates Hepatocellular Carcinoma Response to Therapy by Altering the P53-dependent Cell Death Pathway. J. Exp. Clin. Cancer Res. 38 (1), 252. 10.1186/s13046-019-1246-4 31196136 PMC6567523

[B44] Zhao Y. Ye X. Chen R. Gao Q. Zhao D. Ling C. (2020). Sirtuin 7 Promotes Non-small C-ell L-ung C-ancer P-rogression by F-acilitating G1/S P-hase and E-pithelial-mesenchymal T-ransition and A-ctivating AKT and ERK1/2 S-ignaling. Oncol. Rep. 44 (3), 959–972. 10.3892/or.2020.7672 32705247 PMC7388485

