# Supplementary material for: Discovery of SIRT7 Inhibitor as New Therapeutic Options Against Liver Cancer
Source: Front Cell Dev Biol. 2022 Jan 31;9:813233. doi: 10.3389/fcell.2021.813233 (PMC8841758; doi:10.3389/fcell.2021.813233)
Supplement: Supplementary file 1 [file DataSheet1.docx]

**Figure S1. Ramachandran plot analysis for the predicted SIRT7 structure.** The predicted structural model of SIRT7 was used as input, Ramachandran plot was obtained from PyMod3(Janson and Paiardini, 2021).The sum of the residues located in the most favored regions (75.4%) and residues in additional allowed regions (16.3%) is 91.7%. The residues in the allowable regions account for more than 90% of the entire protein.


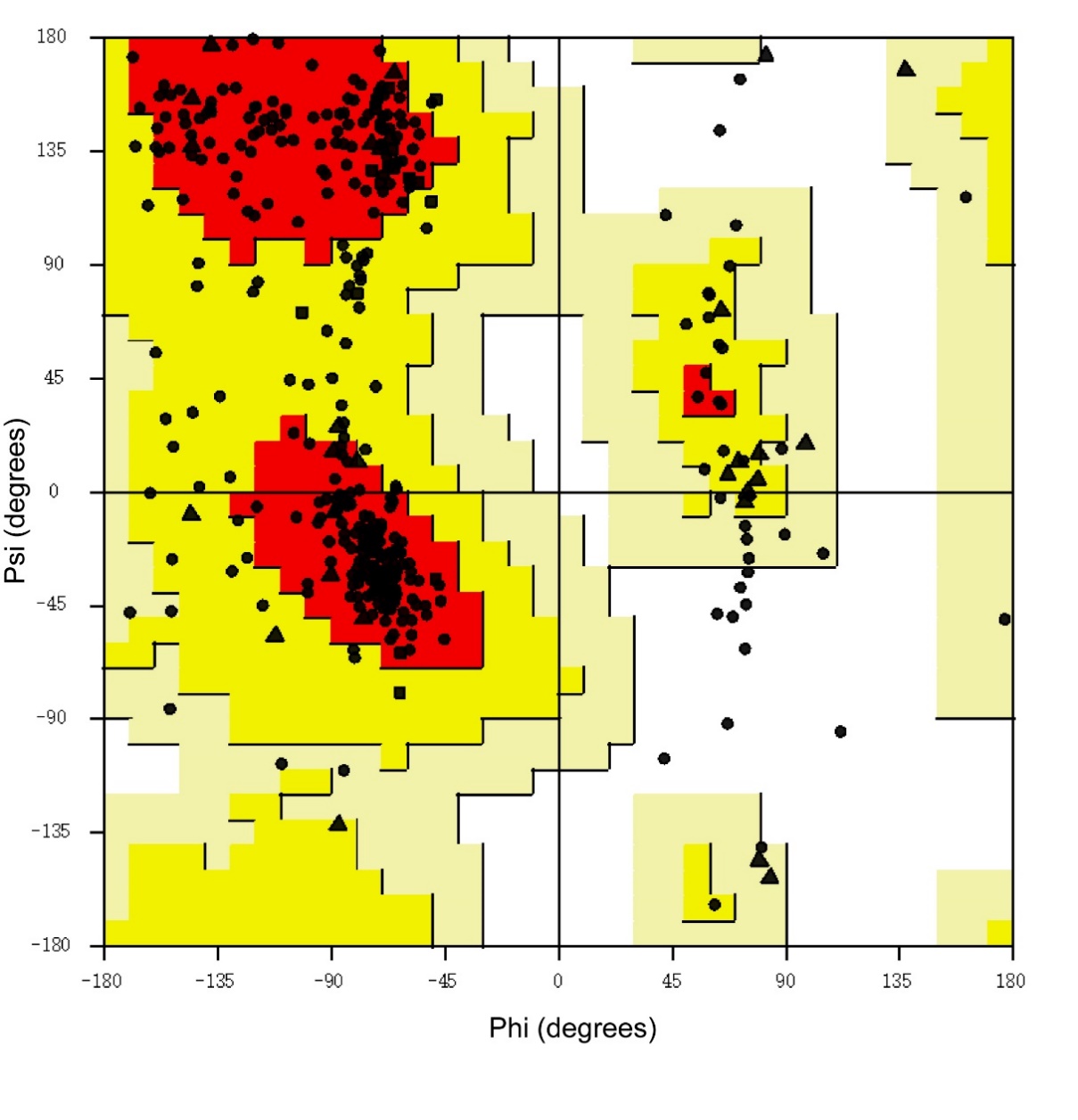


| Non-GLY and non-PRO residues | 349 |
| --- | --- |
| Residues in the most favoured regions | 263 (75.4%) |
| Residues in additional allowed regions | 57 (16.3%) |
| Residues in generously allowed regions | 18 (5.2%) |
| Residues in disallowed regions | 11 (3.2%) |

**Figure S2. 2800Z and 40569Z inhibit growth of HepG2 cell but show low toxicity to L02 cells.** HepG2 (A) and L02 (B) cells were treated with varies of compounds with concentrations as indicated and cell viabilities were detected by using CCK8 assay. Both 2800Z and 40569Z showed ability of inhibiting the growth of HepG2 cells but low toxicity to L02 cells (B). In contrast, 86866Z failed to inhibit the growth of HepG2 cells and showed toxicity to L02 cells.


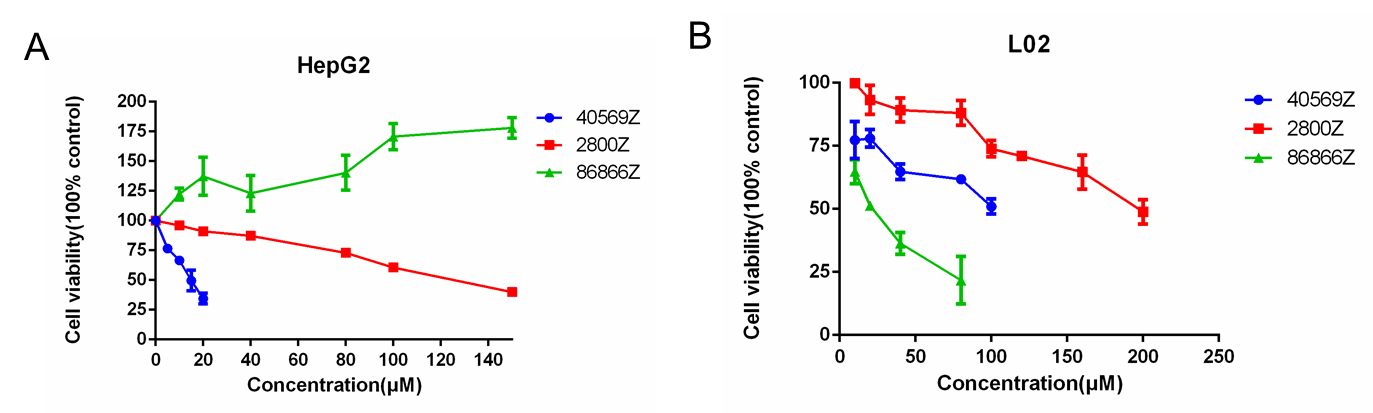


**Figure S3. Synergy analysis between compounds and sorafenib**. Synergy between these compounds and sorafenib were assessed based on synergy finder (Ianevski et al., 2020).The synergy score of 2800Z and sorafenib was 4.793 (A) while 40569Z and sorafenib was 6.168 (B), which indicated the interaction between between compounds and sorafenib were likely to be additive.


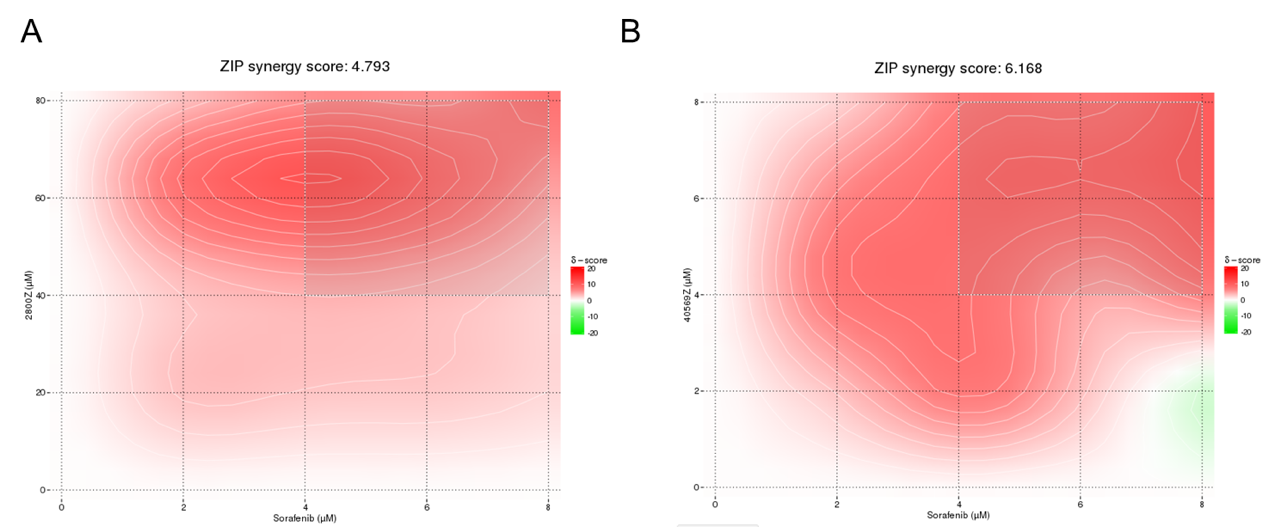


**Figure S4.** HPLC purity analysis of 40569Z (A)and 2800Z(B).


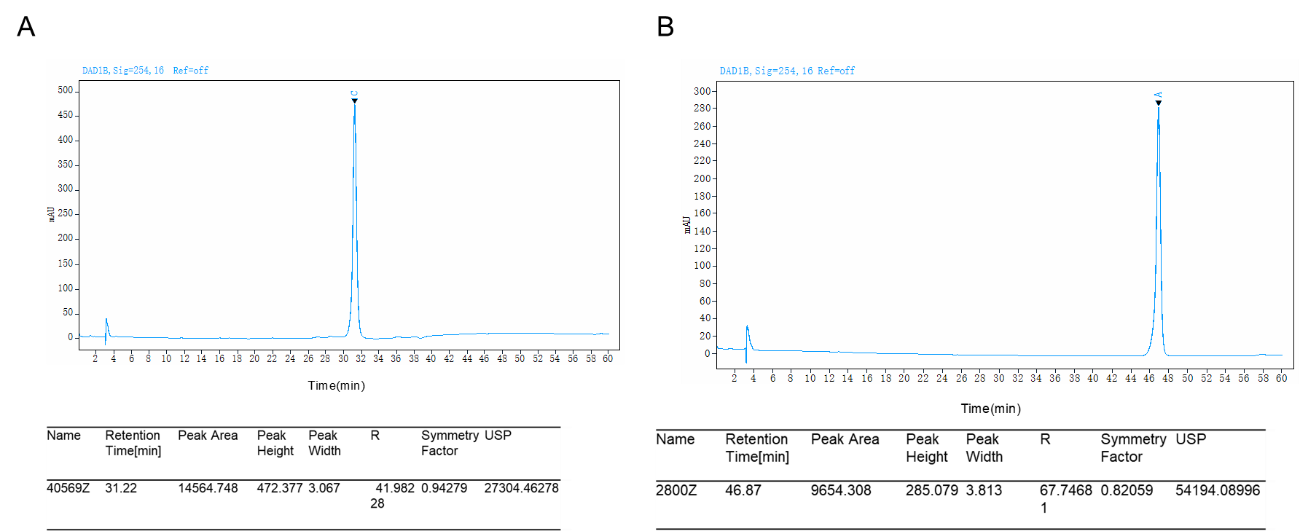


**Supplementary references:**

Ianevski, A., Giri, A.K., and Aittokallio, T. (2020). SynergyFinder 2.0: visual analytics of multi-drug combination synergies. *Nucleic Acids Research* 48(W1)**,** W488-W493. doi: 10.1093/nar/gkaa216.

Janson, G., and Paiardini, A. (2021). PyMod 3: a complete suite for structural bioinformatics in PyMOL. *Bioinformatics* 37(10)**,** 1471-1472. doi: 10.1093/bioinformatics/btaa849.
